# Exaggerated inflammatory response to bacterial products in decompensated cirrhotic patients is orchestrated by interferons IL-6 and IL-8

**DOI:** 10.1152/ajpgi.00012.2022

**Published:** 2022-02-23

**Authors:** Victoria T. Kronsten, Charlotte A. Woodhouse, Ane Zamalloa, Tiong Yeng Lim, Lindsey A. Edwards, Marc Martinez-Llordella, Alberto Sanchez-Fueyo, Debbie L. Shawcross

**Affiliations:** Institute of Liver Studies, Department of Inflammation Biology, School of Immunology and Microbial Sciences, Faculty of Life Sciences and Medicine, King’s College London, London, United Kingdom

**Keywords:** cirrhosis-associated immune dysfunction, decompensated cirrhosis, IL-6, IL-8, interferons

## Abstract

Cirrhosis-associated immune dysfunction (CAID) contributes to disease progression and organ failure development. We interrogated immune system function in nonseptic compensated and decompensated cirrhotic patients using the TruCulture whole blood stimulation system, a novel technique that allows a more accurate representation than traditional methods, such as peripheral blood mononuclear cell culture, of the immune response in vivo. Thirty cirrhotics (21 decompensated and 9 compensated) and seven healthy controls (HCs) were recruited. Whole blood was drawn directly into three TruCulture tubes [unstimulated to preloaded with heat-killed *Escherichia coli* 0111:B4 (HKEB) or lipopolysaccharide (LPS)] and incubated in dry heat blocks at 37°C for 24 h. Cytokine analysis of the supernatant was performed by multiplex assay. Cirrhotic patients exhibited a robust proinflammatory response to HKEB compared with HCs, with increased production of interferon-γ-induced protein 10 (IP-10) and IFN-λ1, and to LPS, with increased production of IFN-λ1. Decompensated patients demonstrated an augmented immune response compared with compensated patients, orchestrated by an increase in type I, II, and III interferons, and higher levels of IL-1β, IL-6, and IL-8 post-LPS stimulation. IL-1β, TNF-α, and IP-10 post-HKEB stimulation and IP-10 post-LPS stimulation negatively correlated with biochemical markers of liver disease severity and liver disease severity scores. Cirrhotic patients exposed to bacterial products exhibit an exaggerated inflammatory response orchestrated by IFNs, IL-6, and IL-8. Poststimulation levels of a number of proinflammatory cytokines negatively correlate with markers of liver disease severity raising the possibility that the switch to an immunodeficient phenotype in CAID may commence earlier in the course of advanced liver disease.

**NEW & NOTEWORTHY** Decompensated cirrhotic patients, compared with compensated patients, exhibit a greater exaggerated inflammatory response to bacterial products orchestrated by interferons, IL-6, and IL-8. Postbacterial product stimulation levels of a number of pro-inflammatory cytokines negatively correlate with liver disease severity biomarkers and liver disease severity scores raising the possibility that the switch to an immunodeficient phenotype in cirrhosis-associated immune dysfunction may commence earlier in the course of advanced liver disease.

## Graphical abstract

**Figure:**
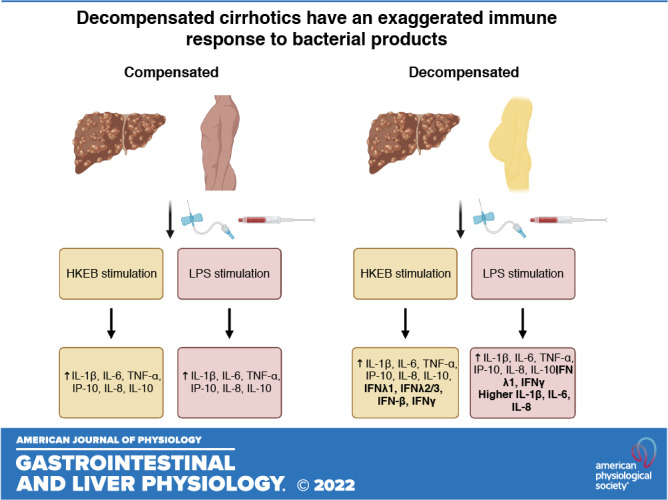

## INTRODUCTION

Cirrhosis-associated immune dysfunction (CAID) describes the wide array of abnormalities observed in the immune system of cirrhotic patients. It encompasses both overt systemic inflammation and acquired immunodeficiency with impaired response to pathogens (1). CAID plays a pivotal role in the development of disease progression, acute-on-chronic liver failure (ACLF), and organ failure and increases the propensity to infection (1–5). The natural history of cirrhosis progresses from an asymptomatic phase termed compensated cirrhosis, to the development of complications from portal hypertension and hepatic insufficiency, named decompensated cirrhosis (6). CAID is dynamic and occurs across the spectrum of all etiologies of cirrhosis; over time the immune response switches from a proinflammatory response in compensated cirrhosis to an anti-inflammatory compensatory (immunodeficient) response in decompensated disease and ACLF (7, 8).

At the smaller end of the scale, patients with compensated and stable decompensated cirrhosis exhibit low-grade systemic inflammation with greater production of proinflammatory cytokines (5) by activated circulating and tissue-resident immune cells (4). Systemic inflammation, in the absence of infection, is pathognomonic of CAID and is instigated by bacterial translocation from the gut to the systemic circulation (9). ACLF patients exhibit the most extreme immune disturbances, with an immune system phenotype characterized by severe immunodeficiency, with a reduced innate and adaptive immune response against pathogens, which increases the risk of bacterial infection (8), accompanied by high-grade systemic inflammation (4). The compensatory anti-inflammatory response syndrome (CARS), characterized by decreased monocyte expression of human leukocyte antigen-DR (HLA-DR), reduced synthesis of proinflammatory cytokines, and increased expression of anti-inflammatory cytokines such as IL-10 (2, 8, 10), is central to this immunodeficient state.

Bacterial infections are frequent in cirrhosis, carry high morbidity and mortality, trigger acute decompensation (3), and are the most common precipitant for ACLF development (accounting for 30% of cases) (11). Thus the immune response of cirrhotic patients to bacteria and their products is of utmost importance.

Many studies have been performed looking at the dysregulated and malfunctioning immune system in patients with compensated and decompensated cirrhosis, acute liver failure, and ACLF (2, 5, 8, 12, 13). However, typically human innate and adaptive immune responses are studied ex vivo/in vitro in a compartmentalized fashion using peripheral blood mononuclear cells (PBMCs) or isolated immune cell subsets. Thus the contribution of mediators such as platelets, granulocytes, circulating miRNA, and extracellular vesicles is not captured when using isolated immune subsets to study induced immune responses. The composition of the circulating blood compartment significantly alters as the severity of liver disease worsens, notably with thrombocytopaenia (14). Beyond their role in hemostasis, platelets interact with leucocytes and endothelial cells (15) and express Toll-like receptors (TLRs), enabling them to recognize pathogen-associated molecular patterns (PAMPs) (16). The omission of the contribution of platelets to the immune response in cirrhosis is therefore of importance when studying CAID.

Isolating PBMCs to study immune response involves transportation of collected blood to the laboratory and subsequent Ficoll-Hypaque gradient centrifugation (17). Stimulation can then be performed instantaneously, or isolated PBMCs can be cryopreserved to allow batch analysis at a later date (18). Such processes involve the risk of sample contamination; sample handling can result in an inconsistent loss of cells and cryopreservation can affect functional responsiveness (19).

The use of whole blood assays to test immune responses minimizes sample handling steps; it also maintains total leucocytes, platelets, and other blood components in the sample and may therefore present a more accurate picture of the immune response to stimulation (20, 21). Studies comparing induced responses using whole blood assays and PBMCs have found striking differences in results, with higher levels of nonspecific cytokines observed in PBMC cultures compared with whole blood (22). Furthermore, whole blood assays provide a more reproducible approach than PBMC culture for immunophenotyping studies (22).

We aimed to determine if there was evidence of immune system dysfunction in nonseptic stable cirrhotic patients, and if this was related to liver disease severity, using a novel technique that has not previously been used in cirrhosis: the TruCulture whole blood stimulation system. TruCulture tubes were used to assess immune response by measuring cytokine secretion at baseline and after incubation with heat-killed *Escherichia coli* 0111:B4 (HKEB) [stimulates Toll-like receptor 2 (TLR2) and Toll-like receptor 4 (TLR4) production] and lipopolysaccharide (LPS/endotoxin) (stimulates TLR4 production). Though still ex vivo, the TruCulture procedure uses whole blood, minimizing contamination and sample handling, and captures immune cell activity at the time and place of sample collection preserving physiological cellular interactions (18).

## PATIENTS AND METHODS

### Study Design and Inclusion Criteria

An observational cross-sectional study was performed. Thirty patients with cirrhosis (22 males and 8 females) were recruited from the Liver Unit at King’s College Hospital, London.

Patients who were between 18 and 75 yr old with a clinical, radiological, or histological diagnosis of any etiology of cirrhosis and who had capacity to consent to the study were recruited. Patients with cirrhosis caused by viral hepatitis were only included if they were hepatitis B virus (HBV) DNA or hepatitis C virus (HCV) RNA negative.

Seven nonsmoking healthy controls (HCs) (22 males, 8 females), without a history of liver disease, were also recruited.

### Exclusion Criteria

Patients were excluded if they had been treated for active variceal bleeding, bacterial infection, spontaneous bacterial peritonitis, overt hepatic encephalopathy, hepatorenal syndrome, organ failure, or ACLF within the previous 14 days. Patients were also excluded if there was suspicion or evidence of current infection, antibiotic use within the past 14 days (excluding rifaximin for chronic hepatic encephalopathy), liver transplantation, hepatic or extrahepatic malignancy, active inflammatory disease, severe renal impairment (creatinine >150 µmol/L), or known immunodeficiency, e.g., HIV. Patients who were pregnant or breastfeeding were excluded. Patients who had received more than 2 wk of immunosuppressive treatment within 8 wk of recruitment were also excluded.

### Definition of Compensated and Decompensated Cirrhosis

Decompensated cirrhosis was defined by the presence of ascites and/or hepatic encephalopathy and/or jaundice (serum bilirubin >51.3 µmol/L). All decompensated patients had stable chronic decompensation and did not have acute decompensation (as per CANONIC definition) (11). Patients without these aforementioned characteristics were deemed compensated.

### Consent and Data Collection

This study was performed in accordance with the Declaration of Helsinki. Ethical approval was granted by the North of Scotland Research Ethics Committee REC (15/NS/0062). After fully informed written consent was obtained, clinical, biochemical and physiological data were collected. Model for end-stage liver disease (MELD) score (23), United Kingdom Model for End-Stage Liver Disease (UKELD) score (24), and Child-Pugh Grade (25) were also calculated.

### Sample Collection

One milliliter of whole blood was drawn from each consented patient/HC into three separate TruCulture tubes (Myriad RBM, Austin, TX) (https://rbm.q2labsolutions.com/products-services/truculture/#tc-stimulants). TruCulture tubes were null/unstimulated or preloaded with HKEB or LPS.

As described by Duffy et al. (18), all tubes were then incubated in dry heat blocks at 37°C (±1°C) room air for 24 h (±15 min). After incubation the tubes were opened and a mechanical separator (valve) was inserted to separate the supernatant from sedimented cells and to stop the stimulation reaction. The supernatant was aliquoted and immediately frozen at −80°C in an upright position until further analysis.

Cytokine analysis on the supernatants from the TruCulture whole blood stimulation system [measuring interleukin-1β (IL-1β), interleukin-6 (IL-6), tumor necrosis factor-α (TNF-α), interferon-γ-induced protein-10 (IP-10), interferon-λ1 (IFN-λ1), interleukin-8 (IL-8), interferon-α2 (IFN-α2), interferon-λ2/3 (IFN-λ2/3), interferon-β (IFN-β), interleukin-10 (IL-10), and interferon-γ (IFN-γ)] was performed in batch using a commercially available predefined multiplex assay [Human Anti-Virus Response Panel LEGENDplex (740390), BioLegend, San Diego, CA] (https://www.biolegend.com/en-us/products/legendplex-human-anti-virus-response-panel-13-plex-with-v-bottom-plate-12748?Clone=) and read on the flow cytometer (BD LSR Fortessa, BD Biosciences, San Diego, CA). Samples were run in duplicate.

### Statistical Analysis

The threshold of detection was assigned as the determined value for cytokine values that were above or below the detection limit.

Statistical analysis was performed using GraphPad Prism 9.3.0 for Mac (GraphPad Software, San Diego, CA). Data are presented as number, percentages, or medians with corresponding 25th and 75th percentiles (interquartile range). Comparisons of nonparametric continuous variables between two groups were made with the Mann-Whitney *U* test, or the Wilcoxon signed-rank test for paired continuous variables. The Kruskal-Wallis test was used for comparisons of nonparametric continuous variables between three or more groups. Nominal variables were compared using the χ^2^-test. Spearman’s rank correlation test was used for the assessment of association between nonparametric variables. All tests were two-tailed, and significance was accepted as *P* < 0.05.

## RESULTS

Thirty patients with cirrhosis were recruited and compared with seven healthy controls (HCs). Levels of IL-1β, IL-6, TNF-α, IP-10, IFN-λ1, IL-8, IFN- α2, IFN-λ2/3, IFN-β, IL-10, and IFN-γ were measured from unstimulated/null TruCulture supernatant tubes, and after HKEB and LPS stimulation. Median age was 37 yr (interquartile range: 33–52) in the HCs, and 56.5 yr (47.75-66) (*P* = 0.02) in the patient group. There was no significant difference in sex distribution between HCs (29% female) and patients (27% female) (*P* = 0.92). Patient characteristics are summarized in Table 1.

**Table 1.** Patient characteristics

	*A*: Patients (*n* = 30)	*B*: Compensated (*n* = 9)	*C*: Decompensated* (*n* = 21)	*B* vs. *C P* Value
Age, yr	56.5 (47.75–66)	58 (49–66)	53 (45.5–67)	NS
Female sex	8 (27%)	2 (22%)	6 (29%)	NS
Etiology of cirrhosis	ALD 17 (57%) NASH 9 (30%) HCV 3 (10%) PBC 1 (3%)	ALD 6 (67%) NASH 2 (22%) HCV 1 (11%)	ALD 11 (52%) NASH 7 (33%) HCV 2 (9%) PBC 1 (5%)	NS
Bilirubin, µmol/L	26 (18.75–46.25)	21 (15.5–25.5)	43.0 (20.5–54)	<0.05
Albumin, g/dL	36.5 (33.0–39.5)	37 (34.5–43.5)	36 (32.5–39)	NS
INR	1.38 (1.19–1.51)	1.26 (1.16–1.34)	1.43 (1.3–1.7)	<0.01
Creatinine, µmol/L	63 (56.25–84)	60 (53.5–74)	65 (57–88)	NS
Total WBC, ×10^9^/L	4.68 (3.73–6.59)	4.58 (4.10 – 4.99)	4.69 (3.56–6.72)	NS
Hepatic encephalopathy	11 (37%)	0 (0%)	11 (52%)	<0.01
Ascites	13 (43%)	0 (0%)	13 (62%)	<0.01
Jaundice	7 (23%)	0 (0%)	7 (33%)	<0.05
MELD score	12 (10-15)	10 (10–11.5)	14 (12–15.5)	<0.001
UKELD score	51 (48.75–54)	50 (47–50.5)	52 (50–55)	<0.01
Child Pugh score	7 (6–9)	5 (5–6)	8 (7–9)	<0.0001
Rifaximin use†	10 (33%)	0 (0%)	10 (48%)	<0.05

Characteristics of patient study group (*n* = 30) (*A*), compensated (*n* = 9) (*B*), and decompensated (*n* = 21) (*C*). Data are quoted as number (*n*) and percentage (%) or median (interquartile range). The Mann-Whitney *U* test was used to compare continuous variables between the two groups. The χ^2^-test was used to compare nominal variables between the two groups. *P* < 0.05 was treated as significant. *Decompensation defined by presence of ascites and/or hepatic encephalopathy and/or jaundice (bilirubin >51.3 µmol/L). All decompensated patients had stable chronic decompensation. †Current rifaximin use or within 1 mo. ALD, alcohol-related liver disease; NASH, nonalcoholic steatohepatitis; HCV, hepatitis C; PBC, primary biliary cholangitis; INR, International normalized ratio; MELD, model for end stage liver disease; UKELD, United Kingdom model for End-Stage liver disease; WBC, white blood cell; NS, not significant.

### Baseline Inflammatory Profiles of HCs and Patients Were Similar

Cytokine levels from unstimulated conditions for HCs and patients are shown in Supplemental Table S1 (all Supplemental material is available at https://doi.org/10.6084/m9.figshare.18130796.v1). IL-10 trended toward being higher in HCs [19.4 pg/ml (14.69–27.02)] than patients [11.02 pg/ml (7.49–21.55)] (*P* = 0.058). Otherwise, no significant differences were seen in the cytokine profile of HCs and the patient group.

### Cirrhotic Patients Had an Exaggerated Response to HKEB and LPS Stimulation

Cytokine levels post-HKEB and LPS stimulation for HCs and patients are shown in Supplemental Table S1 (https://doi.org/10.6084/m9.figshare.18130796.v1).

### Response to HKEB

**Figure 1.**
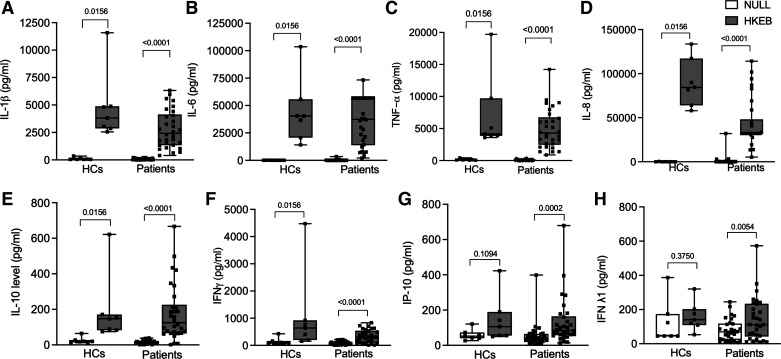
Cirrhotic patients had an exaggerated immune response to HKEB with a significant increase in IP-10 and IFN-λ1 not seen in HCs. Cytokine levels in null and HKEB-stimulated conditions in HCs (*n* = 7, 5 males and 2 females) and cirrhotic patients (*n* = 30, 22 males and 8 females). *A*: IL-1β levels in null and HKEB-stimulated conditions in HCs and patients. *B*: IL-6 levels in null and HKEB-stimulated conditions in HCs and patients. *C*: TNF-α levels in null and HKEB-stimulated conditions in HCs and patients. *D*: IL-8 levels in null and HKEB-stimulated conditions in HCs and patients. *E*: IL-10 levels in null and HKEB-stimulated conditions in HCs and patients. *F*: IFN-γ levels in null and HKEB-stimulated conditions in HCs and patients. *G*: IP-10 levels in null and HKEB-stimulated conditions in HCs and patients. *H*: IFN-λ1 levels in null and HKEB-stimulated conditions in HCs and patients. Units are pg/mL for all values. For box-and-whisker plots: midline, median; perimeters, 25th to 75th centile; whiskers, minimum to maximum values. Individual data points are denoted by a square symbol. Wilcoxon-matched-pairs signed rank test was used for statistical analysis. *P* < 0.05 was treated as significant. HC, healthy controls; HKEB, heat-killed *Escherichia coli* 0111:B4; IL-1β, interleukin-1β; IL-6, interleukin-6; TNF-α, tumor necrosis factor-α; IL-8, interleukin-8; IL-10, interleukin-10; IFN-γ, interferon-γ; IP-10, interferon-γ-induced protein-10; IFN-λ1, interferon-λ1.

A significant increase in IL-1β, IL-6, TNF-α, IL-8, IL-10, and IFN-γ levels was seen with HKEB-stimulated tubes compared with matched null tubes for both HCs and patients (Fig. 1 and Supplemental Table S1).

A significant increase in IP-10 and IFN-λ1 was seen in the HKEB-stimulated tubes [84.81 pg/ml (58.77–164.5) and 112.4 pg/ml (41.94–233.7), respectively] compared with the null tubes [42.52 pg/ml (27.08–64.62)] and 68.94 pg/ml (22.64–117.6), respectively] (*P* = 0.0002 and *P* = 0.005, respectively) for patients, but this increase was not seen in HCs (Fig. 1). IFN-β levels trended toward being higher in the HKEB-stimulated tubes [118.7 pg/ml (65.85–317.2)] compared with matched null tubes [65.85 pg/ml (12.87–125.4)] for patient samples (*P* = 0.057), but this response was not seen in HCs. No changes post-HKEB stimulation in IFN-α2 levels nor IFN-λ2/3 levels were seen in patients nor controls.

### Response to LPS

**Figure 2.**
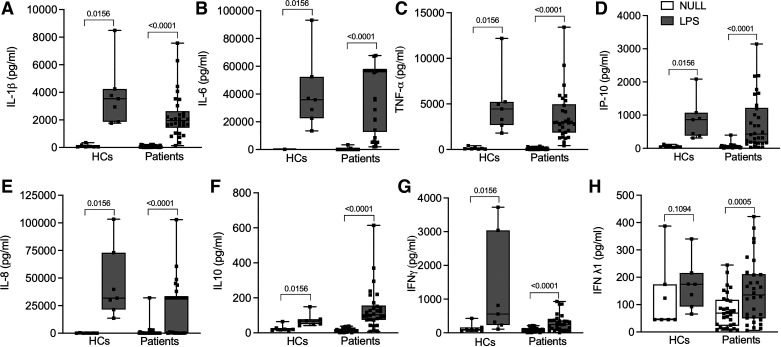
Cirrhotic patients had an exaggerated immune response to LPS with a significant increase in IFN-λ1 not seen in HCs. Cytokine levels in null and LPS-stimulated conditions in HCs (*n* = 7, 5 males and 2 females) and cirrhotic patients (*n* = 30, 22 males an 8 females). *A*: IL-1β levels in null and LPS-stimulated conditions in HCs and patients. *B*: IL-6 levels in null and LPS-stimulated conditions in HCs and patients. *C*: TNF-α levels in null and LPS-stimulated conditions in HCs and patients. *D*: IP-10 levels in null and LPS-stimulated conditions in HCs and patients. *E*: IL-8 levels in null and LPS-stimulated conditions in HCs and patients. *F*: IL-10 levels in null and LPS-stimulated conditions in HCs and patients. *G*: IFN-γ levels in null and LPS-stimulated conditions in HCs and patients. *H*: IFN-λ1 levels in null and LPS-stimulated conditions in HCs and patients. Units are pg/mL for all values. For box-and-whisker plots: midline, median; perimeters, 25th to 75th centile; whiskers, minimum to maximum values. Individual data points are denoted by a square symbol. Wilcoxon matched pairs signed rank test was used for statistical analysis. *P* < 0.05 was treated as significant. HC, healthy controls; LPS, lipopolysaccharide; IL-1β, interleukin-1β; IL-6, interleukin-6; TNF-α, tumor necrosis factor-α; IP-10, interferon-γ-induced protein-10; IL-8, interleukin 8; IL-10, interleukin-10; IFN-γ, interferon-γ; IFN-λ1, interferon-λ1.

A significant increase in levels of IL-1β, IL-6, TNF-α, IP-10, IL-8, IL-10, and IFN-γ was seen with the LPS-stimulated tubes compared with matched null tubes for both HCs and patients (Fig. 2) (Supplemental Table S1).

**Figure 3.**
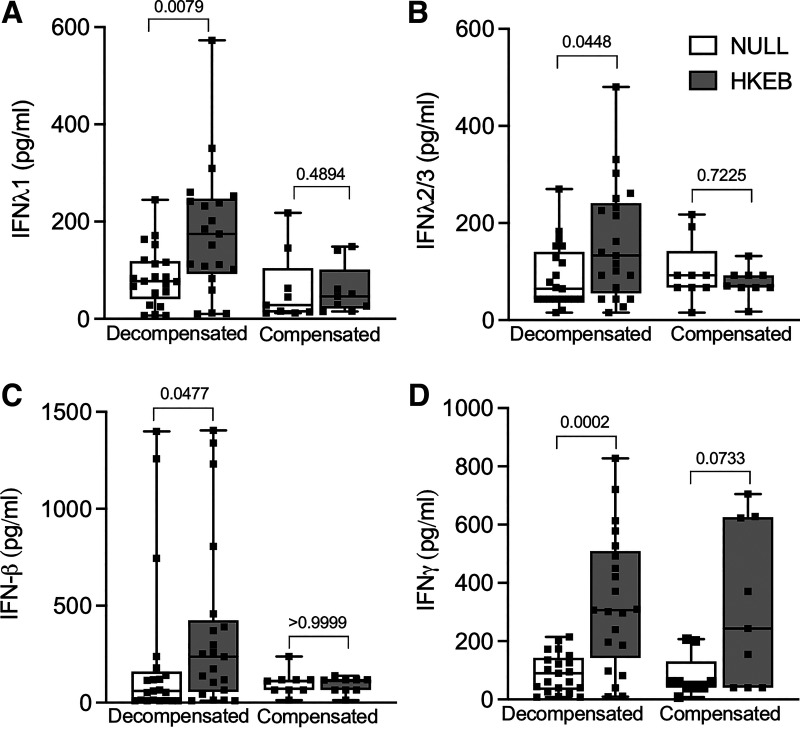
IFN-λ1, IFN-λ2/3, IFN-β, and IFN-γ levels in null and HKEB-stimulated conditions in decompensated and compensated patients (*n* = 21, 15 males and 6 females) and compensated (*n* = 9, 7 males and 2 females) cirrhotic patients. *A*: IFN-λ1 levels in null and HKEB-stimulated conditions in decompensated and compensated patients. *B*: IFN-λ2/3 levels in null and HKEB-stimulated conditions in decompensated and compensated patients. *C*: IFN-β levels in null and HKEB-stimulated conditions in decompensated and compensated patients. *D*: IFN-γ levels in null and HKEB-stimulated conditions in decompensated and compensated patients. Units are pg/mL for all values. For box-and-whisker plots: midline, median; perimeters, 25th to 75th centile; whiskers, minimum to maximum values. Individual data points are denoted by a square symbol. Wilcoxon matched pairs signed rank test was used for statistical analysis. *P* < 0.05 was treated as significant. HKEB, heat-killed *Escherichia coli* 0111:B4; IFN-λ1, interferon-λ1; IFN-λ2/3, interferon-λ2/3; IFN-β, interferon-β; IL-1β, interleukin-1β; IFN-γ, interferon-γ.

A significant increase in IFN-λ1 was seen in the LPS-stimulated tubes [135 pg/mL (49.69–211.6)] compared with the null tubes [68.94 pg/mL (22.64–117.6)] in patients (*P* = 0.0005), but this increase was not seen in HCs (Figs. 2 and 3). IFN-α2 levels trended to being higher post-LPS stimulation [11.83 pg/mL (9.01–25.83)] compared with matched null tubes [9.12 pg/mL (8.68–12.96)] in patients (*P* = 0.058), but this increase was not seen in HCs. No changes post-LPS stimulation in IFN-β nor IFN-λ2/3 levels were seen in neither patients nor controls.

Absolute IL-10 levels trended to being higher post-LPS stimulation in patients [103.5 pg/mL (74.09–156.3)] than HCs [66.12 pg/mL (49.69–78.11)] (*P* = 0.08).

### Decompensated Cirrhotic Patients Exhibited a Strong Inflammatory Response to HKEB and LPS Demonstrated by IFNs, IL-1β, IL-6, and IL-8

Patient characteristics are summarized in Table 1. All decompensated patients had stable chronic decompensation (11). As expected, decompensated patients had significantly higher biochemical markers of liver disease severity [bilirubin and international normalized ratio (INR)] and higher disease severity scores (MELD, UKELD, and Child Pugh score).

### Response to HKEB

A significant increase in IL-1β, IL-6, TNF-α, IP-10, IL-8, and IL-10 levels was seen post-HKEB stimulation compared with matched null conditions for both compensated and decompensated patients (Supplemental Table S2).

A significant increase in IFN-λ1, IFN-λ2/3, IFN-β, and IFN-γ was seen post-HKEB stimulation compared with matched null conditions in the decompensated patients, but this increase was not seen in the compensated patients (Fig. 3) (Supplemental Table S2).

IFN-λ1 levels post-HKEB stimulation were higher in decompensated [174.7 pg/ml (92.42–247.1)] than compensated patients [46.27 pg/ml (21.11–101.4)] (*P* = 0.01). IFN-λ2/3 levels trended to being higher post-HKEB stimulation in decompensated [133.3 pg/ml (55.02–241.1)] than compensated [70.26 pg/ml (67–92.5)] patients (*P* = 0.08).

### Response to LPS

A significant increase in IL-1β, IL-6, TNF-α, IP-10, IL-8, and IL-10 levels was seen post-LPS stimulation compared with matched null conditions for both compensated and decompensated patients (Supplemental Table S2).

**Figure 4.**
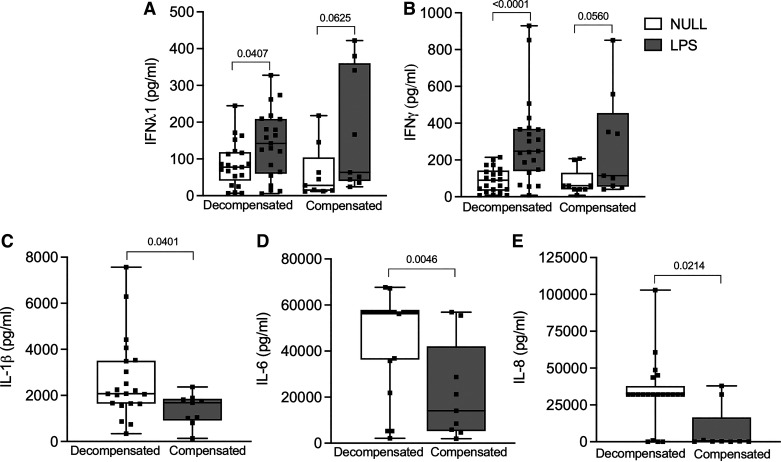
Cytokine levels in null and LPS-stimulated conditions in decompensated (*n* = 21, 15 males and 6 females) and compensated patients (*n* = 9, 7 males, 2 females), post-LPS stimulation and compensated cirrhotic patients, and cytokine levels post-LPS-stimulated conditions. *A*: IFN-λ1 levels in null and LPS-stimulated conditions in decompensated and compensated patients. *B*: IFN-γ levels in null and LPS-stimulated conditions in decompensated and compensated patients. *C*: IL-1β post-LPS stimulation in decompensated and compensated patients. *D*: IL-6 levels post-LPS stimulation in decompensated and compensated patients. *E*: IL-8 levels post LPS stimulation in decompensated and compensated patients. Units are pg/mL for all values. For box-and-whisker plots: midline, median; perimeters, 25th to 75th centile; whiskers, minimum to maximum values. Individual data points are denoted by a square symbol. Wilcoxon matched pairs signed rank test was used for statistical analysis in *A* and *B*. The Mann-Whitney *U* test was used for statistical analysis in *D* and *E*. *P* < 0.05 was treated as significant. LPS, lipopolysaccharide; IFN-λ1, interferon-λ1; IFN-γ, interferon-γ; IL-1β, interleukin-1β; IL-6, interleukin-6; IL-8, interleukin-8.

A significant increase in IFN-λ1 and IFN-γ was seen post-LPS stimulation compared with matched null conditions in the decompensated patients, but this increase was not seen in the compensated patients (Fig. 4) (Supplemental Table S2).

IL-1β levels post-LPS stimulation were higher in decompensated [2,075 pg/mL (1,641–3,508)] than compensated patients [1,683 pg/mL (913–1,853)] (*P* = 0.04). IL-6 levels post-LPS stimulation were also higher in decompensated patients [56.884 pg/mL (36.306–56,884)] than compensated patients [14,066 pg/mL (5,262–42,085)] (*P* = 0.005). IL-8 levels post-LPS stimulation were also higher in decompensated patients [32,026 pg/mL (32,026–37,825)] than compensated patients [258.6 pg/mL (112.2–16,576)] (*P* = 0.02) (Fig. 4).

### Multiple Poststimulation Cytokine Levels Negatively Correlated with Biochemical Markers of Liver Disease Severity and Liver Disease Severity Scores

**Figure 5.**
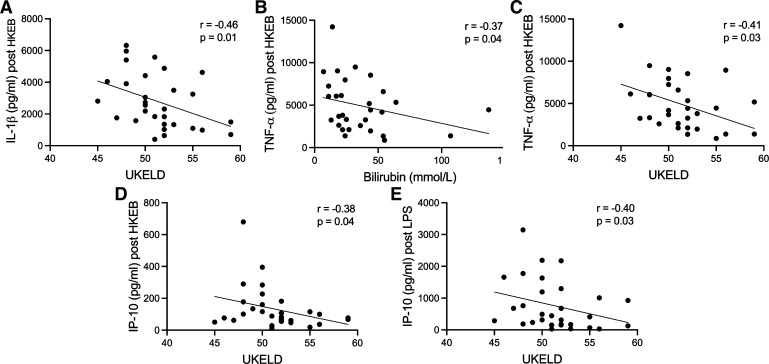
Negative correlation between IL-1β, TNF-α, and IP-10 levels post-*Escherichia coli* 0111:B4 (HKEB) stimulation and IP-10 levels post-LPS stimulation with UKELD and bilirubin in cirrhotic patients (*n* = 30, 22 males and 8 females). *A*: correlation between IL-1β post-HKEB stimulation and UKELD in cirrhotic patients (*r* = −0.46, *P* = 0.01). *B*: correlation between TNF-α post-HKEB stimulation and bilirubin (mmol/L) in cirrhotic patients (*r* = −0.37, *P* = 0.04). *C*: correlation between TNF-α post-HKEB stimulation and UKELD in cirrhotic patients (*r* = −0.41, *P* = 0.03). *D*: correlation between IP-10 post-HKEB stimulation and UKELD in cirrhotic patients (*r* = −0.38, *P* = 0.04). *E*: correlation between IP-10 post-LPS stimulation and UKELD in cirrhotic patients (*r* = −0.4, *P* = 0.03). Spearman’s rank correlation test was used for statistical analysis. *P* < 0.05 was treated as significant. IL-1β, interleukin-1β; TNF-α, tumor necrosis factor-α; IP-10, interferon-γ-induced protein-10; UKELD, United Kingdom Model for End-Stage Liver Disease.

IL-1β levels post-HKEB stimulation in patients negatively correlated with UKELD (*r* = −0.46, *P* = 0.01). TNF-α levels post-HKEB stimulation negatively correlated with bilirubin (*r* = −0.37, *P* = 0.04) and UKELD (*r* = −0.41, *P* = 0.03). IP-10 levels poststimulation negatively correlated with UKELD (*r* = −0.38, *P* = 0.04) (Fig. 5).

IP-10 levels post-LPS stimulation negatively correlated with UKELD (*r* = −0.4, *P* = 0.03).

## DISCUSSION

Systemic inflammation and dysregulation of the immune system are well documented in cirrhosis (5, 7, 13). Trebicka et al. (5) measured baseline plasma levels of 15 markers of systemic inflammation in healthy controls and patients with compensated cirrhosis, acutely decompensated cirrhosis (AD), and ACLF. While most markers were only slightly abnormal in compensated cirrhosis, they were clearly, but still somewhat heterogeneously, raised in AD but markedly elevated in both AD patients who went on to develop ACLF during follow up and in those with ACLF at presentation (5). Solé et al. (13) further confirmed that patients with ACLF exhibit the greatest baseline marked inflammatory state, which correlates with prognosis.

Studies of the immune response to stimulation in cirrhosis have mainly been performed with isolated PBMCs and cell subsets and have produced varying results (26–30). Production of proinflammatory cytokines by PBMCs in response to microbial presence is central to the innate immune response, as these cytokines augment antimicrobial defenses (31). Riordan et al (32) found that monocyte TLR2, but not TLR4, expression was increased in cirrhotic patients (compensated and decompensated) and significantly correlated with serum TNF-α; however, in vitro production of TNF-α by PBMC stimulated by *Staphylococcus aureus* enterotoxin B was significantly blunted. Other studies have revealed increased monocyte TNF-α production to LPS stimulation in both compensated and decompensated patients (26, 27, 30), with a reduction in inflammatory cytokine production (TNF-α, IL-6, IL-1, and IL-12) by myeloid-derived suppressor cells only developing in advanced ACLF (27). Further studies have revealed plasma from acutely decompensated, but not compensated, cirrhotics had reduced TNF-α production in response to LPS stimulation by healthy monocytes, which was mediated by increased plasma prostaglandin E2 related to hypoalbuminaemia (29). Thus the literature is inconclusive, and this may be due to differing laboratory techniques and methodology involving isolating immune cell subsets.

We used a novel whole blood stimulation system to study a heterogenous group of cirrhotic patients, including stable compensated and chronic decompensated patients and discovered four major findings. First, there were minimal differences between the baseline inflammatory profile of HCs and patients. Second, cirrhotic patients had an exaggerated response to HKEB and LPS stimulation. Third, decompensated patients exhibited a robust inflammatory response to HKEB and LPS stimulation demonstrated by a significant rise in interferons (IFNs) and higher levels of IL-1β, IL-6, and IL-8 levels post-LPS stimulation. Neither of these aforementioned findings were observed in compensated patients. Fourth, multiple cytokine levels negatively correlated post-HKEB stimulation and post-LPS stimulation with biochemical markers of liver disease severity and liver disease severity scores.

There was no difference in the baseline inflammatory profile of HCs and the patient group (including decompensated). This is an interesting finding, and not in keeping with previous studies (5, 33). Trebicka et al. (5) only found minimal alterations in baseline inflammatory markers between healthy controls and compensated patients, despite the presence of clinically significant portal hypertension, and other studies have supported this (33). Markers of systemic inflammation have, however, been shown to be markedly raised in decompensated patients at baseline (5, 33). This disparity may well be explained by the reduced liver disease severity in our study, and smaller sample size, however, should be explored in future work. HCs trended to have higher levels of IL-10 than patients. The reason for this is unclear, as multiple studies have demonstrated the opposite findings (12, 34).

HKEB is a heat-killed preparation of *E. coli 0111:B4*, a gram-negative bacterium. Peptidoglycan and LPS on the cell wall stimulate TLR2 and TLR4 production (35), leading to activation of the innate immune system and production of pro-, and anti-inflammatory cytokines. LPS is a component of the outer membrane on gram-negative bacteria and activates TLR4. Downstream signaling leads to an increase in proinflammatory cytokines (36).

An increase in most measured cytokines, demonstrating an activated immune response, was observed in both patients and HCs poststimulation as expected. However, a significant increase in IP-10 and IFN-λ1 was only seen in patients post-HKEB, and a significant increase in IFN-λ1 was only seen in patients post-LPS. IP-10 is a proinflammatory chemotactic cytokine (or chemokine) expressed by a variety of cells including T lymphocytes, natural killer (NK) cells, monocytes, and hepatocytes in response to IFN-γ and thus is central to IFN-induced inflammation. It is a pleiotropic cytokine, promoting chemotaxis of cells expressing its receptor CXCR3 (including T cells, NK cells, macrophages and B cells), cell apoptosis, and angiogenesis (37). These findings clearly demonstrate that stable cirrhotic patients have an increased sensitivity to bacteria and their products, as shown previously in an animal model (38), which leads to an exaggerated innate and adaptive immune response.

The trend toward higher absolute IL-10 levels post-LPS in patients compared with controls (*P* = 0.08) is noteworthy. IL-10, primarily produced by monocytes and lymphocytes, is the principal anti-inflammatory cytokine involved in CARS (10). IL-10 lessens nuclear factor κ-light-chain enhancer of activated B cells activity ultimately leading to decreased secretion of proinflammatory cytokines and also reduces chemokine, platelet-activating factor, and reactive oxygen intermediate production (39). While the stable cirrhotic patients examined are clearly not in the severe immunodeficient stage of CAID, the increase in IL-10 at this stage is noteworthy.

Decompensated patients exhibited an amplified immune response to HKEB and LPS stimulation, with a significant rise in IFNs (IFN-λ1, IFN-λ2/3, IFN-β, and IFN-γ levels post-HKEB stimulation and IFN-λ1 and IFN-γ post-LPS stimulation). Compensated patients did not mount an IFN response, similar to HCs. Furthermore, IFN-λ1 levels post-HKEB stimulation were higher in decompensated than compensated patients. IFN-β is a type 1 IFN. Type 1 IFNs are pleiotropic cytokines, well known for their involvement in the host defense against viruses (40). Their role in host defense against bacteria is less straightforward and can be either beneficial or unfavourable depending on bacterial species (41). Studies investigating the susceptibility of mice to *Listeria* infection have demonstrated that type 1 IFN signaling reduces bacterial clearance, promotes proapoptotic gene expression and augments T-cell sensitivity to apoptotic cell death (42–44). Furthermore, Hackstein et al. (45) demonstrated, in a murine model of liver fibrosis, that failure to control bacterial infection was caused by increased IFN expression from liver myeloid cells, causing myeloid cell IL-10 production weakening antibacterial response. IFN-γ is a type II IFN and, while crucial to both the innate and adaptive immune response, has been associated with autoinflammatory diseases (46) and shown to induce necroptosis, defined as inflammatory cell death, a form of necrosis under conditions such as a massive inflammatory response (47). IFN-λs are type III interferons that are involved in the antiviral response and protect epithelial barrier integrity preventing the transcellular passage of infectious gram-negative bacteria (48).

Decompensated patients had higher levels of IL-1β, IL-6, and IL-8 levels post-LPS stimulation. IL-1β is a proinflammatory cytokine crucial for host defense response (49). IL-6 is necessary for lymphocyte T-helper 17 responses, which impact on neutrophil production and activation. IL-8 stimulates neutrophil recruitment and phagocytosis. Neutrophils then release reactive oxygen species and proteolytic enzymes that contribute to cellular necrosis (4). Neutrophil phagocytic activity and capacity are impaired in cirrhosis, with the most significant dysfunction observed in advanced disease (50). IL-1β, IL-6, and IL-8 levels are also significantly raised in ACLF (13, 51).

Such comparisons between compensated and decompensated patients substantiate that CAID evolves in parallel with liver disease progression (1, 7, 52), with an augmented immune response observed in the decompensated versus compensated patients. This may, in part, be due to the increased expression of pathogen recognition receptors, including TLRs, seen in cirrhotic patients with ascites (32, 53, 54). This demonstrates that the immune system of stable decompensated patients, despite being under almost constant PAMP stimulation due to increased bacterial translocation (55), still exhibits a predominantly proinflammatory phenotype and can mount an innate response, though exaggerated, to pathogens. While this immune response to pathogen presentation is imperative to combat bacterial infection, it is this amplified response, involving significant upregulation of cytokines involved in the innate immune response, that culminates in high-grade systemic inflammation that is characteristic of sepsis-induced ACLF (4). Furthermore, in ACLF this excessive inflammatory response can induce cell apoptosis and necrosis, cellular processes that are related, ultimately, to organ failure (56).

The PREDICT study (57) investigated the clinical course, in parallel with measurement of systemic inflammation, in cirrhotic patients with acute decompensation (AD) to identify predictors of ACLF. Importantly, three different clinical courses of AD with distinct prognoses were identified that corresponded with alterations in systemic inflammation: pre-ACLF, unstable decompensated cirrhosis (UDC), and stable decompensated cirrhosis (SDC). Patients with pre-ACLF exhibited high-grade systemic inflammation at baseline that worsened over time and had the highest mortality, while patients with UDC displayed low-grade systemic inflammation at enrollment, which remained stable throughout follow-up, and patients with SDC exhibited low-grade inflammation at baseline which subsequently improved (57). While our study included patients with stable chronic decompensation, and not AD, it is noteworthy that such changes in systemic inflammation and the immune response were still evident.

IL-1β, TNF-α, and IP-10 post-HKEB stimulation and IP-10 post-LPS stimulation negatively correlated with biochemical markers of liver disease severity and liver disease severity scores. Immune response reprogramming occurs as cirrhosis progresses, likely due to persistent LPS and PAMP stimulation, and manifests as a state of immune paresis (8), similar to that seen in sepsis, and CARS (2). Our finding that the production of several proinflammatory cytokines poststimulation negatively correlates with liver disease severity is in agreement with this switch in CAID phenotype. The immune paresis phenotype has predominantly been noted in ACLF; however our findings suggest that subtle changes impairing immune response to pathogens may occur earlier on in the trajectory of advanced liver disease, though further prospective work is required to substantiate this.

We acknowledge several limitations to our study including the relatively small sample size. This calls for validation in larger studies before wider application of results. The cross-sectional design of the study precludes elucidation of the clinical significance of our findings. It would be beneficial to know which patients went on to deteriorate and develop infections or to develop ACLF and to examine the relationship with their immune responses.

### Conclusions

In cirrhotic patients, the immune response of the unmanipulated circulating compartment to bacterial products is exaggerated when compared with HCs. Furthermore, patients with stable chronic decompensated cirrhosis exhibited a greater immune response than compensated patients, orchestrated by IFNs, IL-6, and IL-8. Poststimulation levels of several proinflammatory cytokines negatively correlated with biochemical markers of liver disease severity and liver disease severity scores in our patient population of stable compensated and decompensated patients, raising the possibility that the switch to an immunodeficient phenotype in CAID may commence earlier in the course of advanced liver disease. Currently, there are inconsistencies in the literature on immune response in cirrhosis, and this may be due to the varying methodology employed. Using whole blood assays to study immune response may give a more accurate depiction of the immune response in vivo by maintaining all components of the circulating compartment.

## SUPPLEMENTAL DATA

10.6084/m9.figshare.18130796.v1 Supplemental Tables 1 and S2: https://doi.org/10.6084/m9.figshare.18130796.v1.

## DATA AVAILABILITY

The datasets generated and analyzed for this study are available on request.

## GRANTS

This study was supported by Phase 1 Integrated Academic Training Research Support funds from King’s College, London (to V.T.K.). This work was performed as part of an National Institute for Health Research academic clinical fellowship (to V.T.K.).

## DISCLOSURES

D.L.S. has participated in consultancy/advisory boards for Norgine Pharmaceuticals, Ltd.; Kaleido Biosciences; Mallinckrodt Pharmaceuticals, Ltd.; EnteroBiotix; ONO Pharma; and Shionogi and has delivered paid lectures for Norgine Pharmaceuticals, Ltd.; Aska Pharmaceutical Co., Ltd.; and Falk Pharma. The remaining authors declare that the research was conducted in the absence of any commercial or financial relationships that could be construed as a potential conflict of interest.

## AUTHOR CONTRIBUTIONS

V.T.K., M.M.-L, A.S.-F, and D.L.S. conceived and designed research; V.T.K., C.A.W., T.Y.L., and L.A.E. performed experiments; V.T.K. and L.A.E. analyzed data; V.T.K. and L.A.E. interpreted results of experiments; V.T.K. prepared figures; V.T.K. drafted manuscript; L.A.E., A.S., and D.L.S. edited and revised manuscript; V.T.K., C.A.W., A.Z., T.Y.L., L.A.E., M.M.-L., A.S.-F., and D.L.S. approved final version of manuscript.
